# In this month’s *Bulletin*

**DOI:** 10.2471/BLT.16.000816

**Published:** 2016-08-01

In the editorial section, Tim Jinks et al. (558) explain why a global response is needed to confront antimicrobial resistance. Melanie M Taylor et al. (559) present global estimates of the penicillin needed to prevent congenital syphilis. 

Andréia Azevedo Soares (562–563) reports on the introduction of *Wolbachia*-infected mosquitoes in Brazil. Rabih El Chammay (564–565) tells Fiona Fleck how the Lebanese health system is improving access to mental health care services.

## Poland

## Asbestosis, mesothelioma and lung cancer

Beata Świątkowska et al. (599–604) report on efforts to provide care for people who worked in asbestos-processing plants.

## Botswana, Ethiopia, Kenya, Lesotho, Malawi, Mozambique, Namibia, Rwanda, South Africa, Swaziland, United Republic of Tanzania, Uganda, Zambia & Zimbabwe

## Tetanus deaths in young men

Shona Dalal et al. (613–621) summarize reports of tetanus deaths and argue for renewed attention to vaccinating adolescent and adult men.

## Sri Lanka

## Surviving pesticide poisoning

Fathima Shihana et al. (622–625) describe a bedside test used to recognize and manage methemoglobinemia.

## Brazil

## Taking services to the patients

Ana Roberta Pati Pascom et al. (626–630) evaluate the provision of peer-administered HIV tests.

## Global

## Consulting with industry

David Stuckler et al. (566–573) analyse versions of WHO’s sugars guidelines.

## Diagnosing Zika infections

Rémi N Charrel et al. (574–584) summarize the information needed for diagnostic test development.

## Using trauma care guidelines

Lacey LaGrone et al. (585–598) review the evidence for references to WHO’s trauma guidelines.

## What people report in surveys

Lisa G Johnston et al. (605–612) insist on the need to check HIV status.

## Improving food quality

LM Neufeld et al. (631–632) call for more evidence to inform nutrition policies.

